# Colorectal cancer in pregnancy: case discussions and real-world data as well as literature review on current knowledge

**DOI:** 10.1093/oncolo/oyaf097

**Published:** 2025-07-07

**Authors:** Suzanne Poulgrain, Ted Gibbons, Cian O’Leary, Sophie Feng, Vikram Jain, Connor Gerard O’Leary

**Affiliations:** Medical Oncology, Mater Hospital, Brisbane, 4101, Australia; Medical Oncology, Mater Hospital, Brisbane, 4101, Australia; Medical Oncology, Mater Hospital, Brisbane, 4101, Australia; Medical Oncology, Mater Hospital, Brisbane, 4101, Australia; Medical Oncology, Mater Hospital, Brisbane, 4101, Australia; Medical Oncology, Mater Hospital, Brisbane, 4101, Australia

**Keywords:** young colorectal cancer, cancer in pregnancy, chemotherapy in pregnancy

## Abstract

Colorectal cancer during pregnancy is a rare event; but given the well-recognized increasing incidence of young-onset colorectal cancer and delayed child-bearing seen in the Western world, it is anticipated to sharply increase in the next two decades. In this paper, the authors analyse three cases of colorectal cancer occurring in pregnancy, including the therapeutic approach and relevant outcomes. Two of the three cases received chemotherapy during pregnancy, all three children were born prematurely with one child having a significantly low birth weight. A review of our institution's referrals related to early-onset colorectal cancer (EOCRC) revealed a 50% increase in incidence in the last decade in line with worldwide trends of increase in this population. This paper also reviews the literature related to EOCRC in pregnancy and current diagnosis and management recommendations. Challenges with the therapeutic approach are experienced when symptoms are masked by the pregnancy, and when the diagnostic workup needs to weigh benefits to the mother with harms to the child. There is a clear and increasing need for consensus guidelines and further research into this field.

**Implications for practice**

Colorectal cancer in pregnancy is likely to be an increasingly frequent clinical scenario given the rising incidence of young-onset colorectal cancer and the trend for delayed childbearing. This article summarises all aspects of care of these patients and current knowledge regarding the safety and risks associated with investigations and treatment of these cancers. Chemotherapy can be considered during the second and third trimester of pregnancy and has been given without significantly increased risk to the developing child in small case series.

## Background

Colorectal cancer is the third most common malignancy worldwide and contributes significantly to cancer-related mortality as the second leading cause of cancer-related deaths worldwide.^1^ Though incidence and outcomes continue to improve in those aged over 50 with colorectal cancer, the same may not necessarily be true of those who develop the disease younger. Early onset colorectal cancer (EOCRC) has various definitions but the most widely utilized is defined as colorectal cancer developing prior to 50 years of age.^2^

Numerous analyses across the last decade have identified a worldwide trend of increasing incidence of EOCRC and seem mostly to be driven by increases in both the USA and Australia, as well as a marked increase in rectal cancer.^3^ In 2019 Liu^4^ extracted data from the International Agency for Research in Cancer and reported a significant increase in EOCRC in 11 of the 12 countries analysed.

Not only is the incidence of EOCRC increasing, but this patient group generally has more advanced disease at diagnosis.^5^ Risk factors for early onset disease include male sex, family history of bowel cancer, obesity and associated metabolic syndromes, smoking, and Western diet patterns.^6^

With a rising incidence in younger people, it is perhaps not surprising that colorectal cancer in pregnancy has become a significant clinical topic in recent years. Presenting symptoms from colorectal cancer can be masked or attributed to those of early pregnancy which can make diagnosis challenging and it has been suggested to contribute to a greater risk of advanced disease at diagnosis.^7,8^ The babies born from mothers with colon cancer have variable clinical outcomes themselves though tend to be favourable, and data suggests chemotherapy during pregnancy can be delivered safely with regards to harm to the fetus.^8^ Rarely cancer in the mother can metastasize to the placenta or fetus but there are no reports of this occurring in the setting of colorectal cancer within a PubMed literature search nor a recent systematic review of this phenomenon.^9^ Given the clinical urgency; delaying treatment until the pregnancy has reached full term may not be clinically possible. Data to guide treatment decisions in this setting is sparse and in need of further development.

Our institution has noted several cases of young women with colorectal cancer diagnosed during pregnancy over the last two years. In this article, we review their cases and outcomes for mother and baby, as well as data collected on all young colorectal cases treated in our network over the last 10 years. The 3 case reports are additionally summarised in Table 1.

**Table 1.** A summary of all three cases of early-onset bowel cancer in pregnancy.

Case	Age	Stage at diagnosis	Gestational age at diagnosis	Treatment while pregnant	Gestational age at delivery (weeks)	Outcome for baby	Outcome for patient
1	35	IV	17/40	FOLFOX for 3 cycles	28/40	Low birth weight NICU support No birth defects	Partial response. RIP due to bowel obstruction Survival 8 months
2	42	IV	16/40	No chemotherapy during pregnancy	28/40	NICU support No birth defects	Stable disease for 8 months prior to progression. RIP due to bowel obstruction at 17 months.
3	35	III	22/40	FOLFOX for 3 cycles	30/40	NICU support No birth defects	Completed early stage treatment Subsequent recurrence with intracranial metastases Survival 18 months

Abbreviations: FOLFOX, fluorouracil oxaliplatin; NICU, Neonatal intensive care unit; RIP, rest in peace;.

## Case 1

Patient 1 was an otherwise well 35-year-old female with a family history of Lynch syndrome, with her mother having passed away from a related colorectal cancer. Her past medical history was significant for psychiatric issues, including post-traumatic stress disorder, generalized anxiety disorder, and depression. In the context of her family history, she had a prior colectomy with ileorectal anastomosis prophylactically, as well as multiple small bowel resections in the context of small bowel obstructions for desmoid tumours. A recent flexible sigmoidoscopy had shown multiple tubular and tubulovillous adenomas; however, she had elected to defer repeat scopes due to pregnancy. This was her second pregnancy following a miscarriage a year prior.

Patient 1 came to our attention when she presented to our emergency department (ED) in late 2021 with right-sided pleuritis whilst 17 weeks pregnant. Initial investigations demonstrated a raised D Dimer of 2.25 mg/L which prompted a computerized tomography (CT) pulmonary angiogram. Aside from a right lung infectious process, this demonstrated new hypodense liver lesions of uncertain aetiology. A magnetic resonance imaging (MRI) scan of her liver demonstrated appearances strongly suggestive of hepatic metastases, with porta hepatis and retroperitoneal lymphadenopathy, and mesenteric nodules at the ileorectal junction. A subsequent liver biopsy confirmed a metastatic colorectal adenocarcinoma, with mismatch repair (MMR) deficiency. Subsequent genomic testing demonstrated an activating Kirsten rat sarcoma (KRAS) G38A mutation. No BRAF (murine sarcoma viral oncogene homolog B) mutation was noted. Her liver function showed signs of progressive deterioration from her original ED presentation, with an alanine aminotransferase (ALT) of 55 U/L, an aspartate aminotransferase (AST) of 255 U/L, a gamma-glutamyl transferase of 77 U/L and an alanine phosphatase of 218 U/L. Bilirubin, albumin, and platelets were within normal limits; however, internationalized normal ratio (INR) was elevated at 1.7, indicating early synthetic liver dysfunction. Carcinogenic embryonic antigen (CEA) was also elevated at 38 µg/L.

The patient was subsequently referred to our oncology clinic for review, where she was first seen at 19 weeks gestation. A decision was made to proceed with doublet chemotherapy with 5-fluorouracil and oxaliplatin (FOLFOX), which commenced in early 2022. Bevacizumab was not co-administered due to concerns for teratogenicity. The patient was also linked closely with the obstetrics team at our hospital for ongoing antenatal care, as well as a diverse multidisciplinary team (MDT) including dietetics, palliative care, psychology, social work, and cancer care coordinators.

Issues arose following the patient's second cycle of chemotherapy with decreased oral intake and malnutrition, as well as significant right upper quadrant pain due to liver capsular stretch. This malnutrition was a multifactorial process involving her intra-abdominal malignancy, her premorbid mental health issues, her pregnancy, and her cancer treatment causing gastrointestinal upset. She was admitted and total parenteral nutrition commenced with dietician support. Severe fetal growth restriction was noted by our obstetric colleagues, with significant concerns for both maternal and fetal well-being and survival. Both the patient and her partner were clear in their wishes that every opportunity be given to protect their baby’s well-being and ensure a safe birth. Following the MDT discussion, a decision was made for elective cesarean section at 28 weeks gestation. The patient’s son was successfully delivered though remained in neonatal intensive care for several months, with significant issues surrounding low birth weight as well as his premature delivery. The baby had no specific birth defects/signs of teratogenicity from chemotherapy. Our patient subsequently had her third cycle of FOLFOX after a 7-week delay due to these issues. Malnutrition and poor weight gain remained significant issues for her. A repeat CT scan after four cycles showed a good treatment response with decreased size of her pelvic mass, mesenteric mass, and diffuse liver metastases. She continued to cycle seven; however, thereafter moved interstate to be closer to family. Shortly after this move, she was admitted to her new local hospital in extremis due to bowel obstruction, ultimately passing away on that admission following a decision for conservative management.

## Case 2:

Our second patient was a 42-year-old female referred to our colleagues in gynae-oncology from obstetrics at 20 weeks gestation with an enlarging right ovarian mass on serial ultrasound (US). Initially noted early in pregnancy, it had increased from 65 mm at 16 weeks gestation to 175mm, prompting referral for further assessment. The patient was otherwise well, with no significant medical history but a family history of colorectal cancer (maternal grandfather, aged 48 at diagnosis). This was her third pregnancy, with two teenage children at home already.

Following discussion at the gynaecology MDT, an MRI pelvis was performed at 24 weeks gestation, which showed another rapid increase in the size of the right ovarian mass to 241mm as well as a 47mm left ovarian mass. Additionally, the MRI demonstrated left external and pelvic lymph nodes, as well as omental deposits concerning for metastases. An omental biopsy returned a positive result for mucinous adenocarcinoma. Serial MRI pelvis demonstrated new liver lesions progressing from prior imaging studies at 25 weeks gestation and showed further progression on dedicated MRI liver at 27 weeks. At this stage, the patient began to clinically deteriorate necessitating admission under obstetrics for pain management, preeclampsia, and renal impairment (non-obstructive). Ultimately, a decision was made for delivery at 28 weeks due to this declining clinical state and rapid progression of her malignancy and the patient underwent cesarean section, hysterectomy, bilateral salpingo-oophorectomy, and omentectomy. Her baby boy was transferred to the neonatal intensive care unit for ongoing care.

Once recovered and discharged from her operation, she was followed up in our oncology clinic. Histology from her operation demonstrated metastatic mucinous adenocarcinoma of extra ovarian origin in her bilateral ovaries, right fallopian tube, and omentum. A KRAS G12D mutation was noted, while BRAF was wild-type. A colonoscopy done two weeks post-delivery confirmed the presence of a cecal primary. We commenced palliative chemotherapy with FOLFOX and bevacizumab approximately 4 weeks post operatively, and she continued this for a total of 10 cycles prior to dropping oxaliplatin for recurrent thrombocytopenia and peripheral neuropathy. She progressed after a further five cycles of fluorouracil and bevacizumab alone and developed painful omental disease necessitating whole pelvic radiation for pain control (22 Gray in five fractions). She had a single cycle of irinotecan prior to admission and subsequent death from bowel obstruction at 17 months from diagnosis. At the time of writing her newborn is doing well and continues to thrive.

## Case 3

Our final patient is a 35-year-old female who presented to ED at 17 weeks gestation with severe constipation, lower back pain, and small-volume rectal bleeding. She had no significant past medical history, and this was her second pregnancy. A colonoscopy performed at 22 weeks demonstrated a large, locally advanced rectal mass (T3) with significant luminal narrowing. Histology from a biopsy taken during this scope demonstrated a rectal adenocarcinoma, with retained MMR staining. A staging non-contrast CT chest and MRI abdomen and pelvis demonstrated a T4b locally advanced lesion with transmural extension 100 mm posteriorly, extramural vascular invasion (EMVI), and involvement of the presacral fascia. No malignant-looking lymph nodes were noted.

Following discussion at the MDT, a plan was made for neoadjuvant chemotherapy while pregnant, given high-risk features (bulky primary, EMVI), with an aim for cesarean section at 32 weeks gestation followed by anterior resection and loop ileostomy, and further adjuvant chemotherapy. FOLFOX was commenced at 23 weeks gestation, with ongoing input from the patient’s private obstetrician. Similar to case 2, this patient’s abdominal pain increased further into her pregnancy. This led to an admission at 30 weeks gestation with increasing pain and discomfort, and a decision was made to deliver her baby at this point via cesarean section. Her baby was born healthy, with no obvious teratogenicity, although did require NICU support for prematurity. A low anterior resection was subsequently performed two weeks post-partum, and chemotherapy recommenced at cycle 4, 3 weeks post-op. Twelve cycles of FOLFOX were completed prior to going onto a program of surveillance and at nine months from diagnosis, only three months into surveillance, a symptomatic brain metastasis was diagnosed. This was treated with craniotomy and post-operative radiation, then a month later a PET detected nodal recurrence. Her care was transferred to another unit so further details are not known but she ultimately passed away at 18 months from diagnosis.

## Summary of case series

Our three cases of colorectal cancer in pregnancy demonstrate valuable clinical insights into this issue. Two of the three patients received chemotherapy while pregnant. Of these, two fetuses were exposed to FOLFOX in utero, both were born premature, and one at a significantly lower birth weight. In the first case, growth retardation was likely not solely due to chemotherapy; multiple issues contributed to malnutrition for the patient, including her active malignancy and significant psychological issues. Neither child exposed to FOLFOX in utero displayed any teratogenic effects at birth. Our experience suggests FOLFOX can be safely used during pregnancy.

All cases required premature delivery, whether due to increasing abdominal pain from a growing fetus and in situ malignancy co-occupying the same limited space in Cases 2 and 3, or malnutrition and growth retardation as in Case 1. This highlights for us the importance of multidisciplinary care for patients like this, with close follow-up from medical oncology, obstetrics, oncological surgery teams, and neonatology to ensure the best outcomes for mother and baby.^10^ With this input, early delivery was successfully performed in all three cases, with all babies being successfully cared for and ultimately discharged home post-partum.

The outcomes for the mothers in these cases highlight a need to treat these cancers as intensively as possible to give young women in these circumstances the best chance of long-term survival.

## Young colorectal cancer: incidence and outcomes from our own institution

Prompted by these notable cases of colorectal cancer in young pregnant women, we elected to audit our institution's data regarding early-onset colorectal cancer cases over the last 10 years. Online records of patients attending all Mater Cancer Care sites across Brisbane, Australia who received treatment for colorectal cancer were identified. Patients under 50 years at diagnosis were selected and were designated as metastatic or early disease. For patients with metastatic disease, overall survival (OS) was calculated from the date of diagnosis to the date of death. For early cases, disease free survival (DFS) was calculated from the date of diagnosis to the date of relapse or last interaction with our service. Data on tumour characteristics and molecular profiling were collected, as were body mass index (BMI) measurements at the initial review. Patients were categorized based on BMI into severely underweight, underweight, normal weight, overweight, or obese categories 1-3 based on World Health Organisation definitions.^11^

## Results

**Figure 1.**
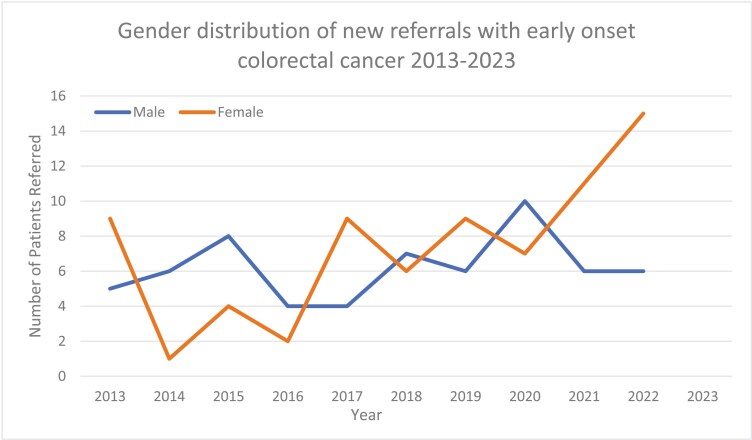
Gender distribution of new cases of early-onset colorectal cancer attending our service 2013-2023.

Between June 2013 and 2023, 135 new patients, 73 female and 62 male, with early onset colorectal cancer received treatment with our service. The incidence of new cases aged less than 50 increased from 14 in 2014 to 21 in 2022 with a predominance of female cases noted in later years (Figure 1).

**Figure 2.**
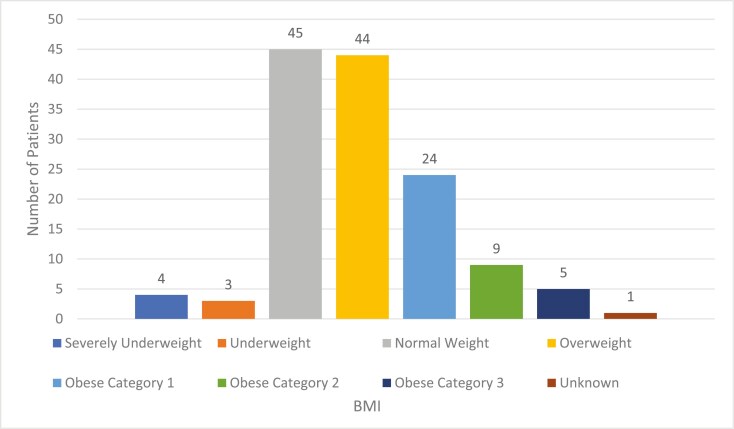
Body Mass Index (BMI) distribution in early-onset colorectal cancer patients at initial oncology review 2013-2023. Obese category 1 as defined by BMI 30-34.9. Obese category 2 is defined as BMI 35-39.9. Obese category 3 is defined as BMI > 40.

The median age of these patients was 42 years old (range 17-50). 60 patients (44%) had metastatic disease at diagnosis. 75 patients (56%) had stage II or III disease, of which 17 patients (23%) developed recurrence; median DFS was 1.5 years (541 days) for those that recurred. The median OS for all patients with metastatic disease (*n* = 76) was two years (734 days). MMR status was available for 81 patients of which seven were deficient (9%). Most patients were overweight or heavier based on BMI at initial review (60.7%, *n* = 82); however, the most represented weight category was “Normal BMI” (*n* = 45, 33.3%) (Figure 2).

## Discussion

Given the observed and recognized increase in the incidence of colorectal cancer among young people,^5^ particularly females,^12,13^ combined with the Western trend of delayed childbearing, we can anticipate an increased presentation of colorectal cancer during pregnancy in the coming decades.

At present there is no mention of pregnancy within the ESMO^14^ nor NCCN^15^ guidelines on colorectal cancer and the specialised clinical practice guideline produced by ESMO on cancer in pregnancy has no mention of bowel cancer^16^ nor does the ASCO article on management of Cancer During pregnancy.^17^ As such there is a need to analyse the unique factors associated with this clinical scenario and come up with consensus guidelines for treatment.

There are a number of risk factors that have potentially been implicated in why colon cancer is on the rise in the young cohort and these include obesity, low physical activity, smoking, higher red meat consumption, and higher fat consumption.

Lynch syndrome generally comprises only 3% of cases of colorectal cancer diagnosed above age 50, in comparison to 10% of cases diagnosed^18^ less than 50. Our clinical data is in line with this with MMR deficiency, the genetic hallmark of Lynch Syndrome found in 8.6% of our cohort. Although Lynch syndrome is enriched in the young cohort, it does not account for most cases, which at present are labelled sporadic.

The diagnosis of colorectal cancer during pregnancy can be fraught with many overlapping symptoms that may delay and mask the ultimate diagnosis. The most common pregnancy symptoms include fatigue (98%), nausea (88%), poor sleep (74%), back pain (60%), vomiting (57%). Symptoms of relevance include^19^ diarrhoea (12%), constipation (32%), and nutritional deficiencies (45%). Rectal bleeding may be attributed to haemorrhoids which occur in 41% of pregnant females^20^ and iron deficiency^21^ in almost 50%. In a systematic review and meta-analysis of red flag symptoms for patients with EOCRC,^22^ the most common symptoms were hematochezia (45%), abdominal pain (40%), and altered bowel habit (27%). Given this crossover in symptoms, a high index of suspicion needs to be maintained. A survey by the Colorectal Cancer Alliance in 2018 found that of the 884 USA patients with colorectal cancer developed less than age 50, 41% waited more than six months prior to seeking medical attention, 75% of patients saw two or more physicians prior to diagnosis and 77% were subsequently diagnosed with stage III or IV disease as a consequence.^23^

Pregnancy is characterized by initial high progesterone and human chorionic gonadotropin (hCG) levels from the corpus luteum and then later by high oestrogen levels.^24^ The unique hormonal milieu of pregnancy can promote growth in numerous benign pathologies including fibroids, dermoids, and hepatic hemangiomas,^25^ but pregnancy has not been shown to be a risk factor for the development of colon cancer and most studies have demonstrated an inverse association between high oestrogen states and the development of colon cancer.^24^

## Diagnostic work up

Many investigations commonly used for abdominal pain, bloating, diarrhoea, and nausea are known to be harmful to developing fetuses and are thus less likely to be ordered, or delayed in their initiation in those known to be pregnant. As a result of these complicating factors, pregnant females are generally diagnosed later in the course of their disease.

There is no clear guideline on the use of colonoscopy during pregnancy published by the Cancer Council of Australia; nor do the Australian Pregnancy Care Guidelines mention their views on colonoscopy. A number of small case series^26,27^ suggest this may be safe in the second trimester but should only be considered for strong indications. Some articles in this field suggest that pregnancy is a relative contraindication to colonoscopy.^28^ Given that the majority, 54%, of young colorectal cancers are diagnosed in the sigmoid and rectum^29^ and that sigmoidoscopy can be performed with light sedation, this represents a safer and more feasible alternative than complete colonoscopy. Case series would suggest this is safe in all trimesters of pregnancy.^30^

Contrast-enhanced MRI, CT, Bone Scan, and positron emission tomography (PET) are not recommended by the *Cancer, Pregnancy, and Fertility: ESMO Clinical Practice Guideline*^16^ which was last updated more than 10 years ago now. Ultrasound of the abdomen and pelvis is recommended and chest X-ray with abdominal shielding exposes a fetus to < 0.01mGy, which carries with it a theoretical increase in childhood cancer of 1:10,000 above baseline risk.^31^ MRI without contrast is generally considered safe during pregnancy, as the magnetic technique used has not been shown to affect the fetus or have affects in early childhood.^32^ Gadolinium, the contrast used in MRI, is known to cross the placenta and be excreted in the urine; although thought to be safe, this has not been proven and there exists a theoretical risk of nephrogenic systemic fibrosis in the developing child due to unbound gadolinium.^32^ A summary of these recommendations in diagnostic workup can be seen in Table 2.

**Table 2.** Diagnostic workup summary.

SAFE Chest X-ray with abdominal shielding US
CONSIDER Sigmoidoscopy CT chest with abdominal shielding MRI without gadolinium PET without CT
AVOID CT Pelvis MRI with gadolinium Colonoscopy

## Treatment

The mainstay of therapy in the absence of pregnancy for colon cancer involves surgery and/or chemotherapy; whereas the mainstay of rectal cancer generally involves chemotherapy and then concurrent chemoradiation followed by surgery; so-called “total neoadjuvant therapy” (TNT). Pelvic radiation is generally never pursued during a viable pregnancy, as this would expose the fetus to teratogenic levels of ionizing radiation.^16^

The drugs most used in the treatment of localized and metastatic colorectal cancer include fluorouracil, oxaliplatin, irinotecan, bevacizumab, and cetuximab; Table 3 summarises the current knowledge specific to their use in pregnancy.

**Table 3.** Summary of systemic agents and knowledge regarding their use in pregnancy.

Drug	Pharmacodynamics	Known or theoretical risks	Conclusions
Fluorouracil	Placental passive diffusion likely. Placenta and breast milk transport in humans has not been documented.^33^ Published reports of placental transfer in mouse and rat studies^34^	Extensively used in case series in 2^nd^-3^rd^ trimester in breast and colorectal cancer.^35-37^ Rates of fetal malformation when used in later trimester 1.2%.^34^ > 30% rate of observed major malformations when used in 1^st^ trimester.^34^ Case reports of micrognathia and hand and foot abnormalities when used in 1^st^ trimester.^38^	Likely safer in 2^nd^ and 3^rd^ trimester To be avoided in 1^st^ trimester
Capecitabine	Placental passive diffusion is likely given animal studies indicate placental transfer^34^	Minimal case series reports of use.	Unknown risk, insufficient data. Suggest avoidance.
Leucovorin	Unknown if excreted in breast milk.	Safe TGA Pregnancy Category A	Safe
Oxaliplatin	90% protein bound.^34^ Pregnancy state is associated with less protein binding ability, hence more free drugs and hence likely increased toxicity.	Several case reports on oxaliplatin (one case of neonatal hypothyroidism in 8 patients treated with FOLFOX for colorectal cancer)^9,34,35^	Relatively safe in 2nd and 3rd trimester.
Irinotecan	No published data on placental transfer in human or animal studies. Theoretical increase in metabolism due to upregulation of hepatic enzymes.^39^	Minimal case series reports of use^40,41^	Unknown risk, insufficient data
Bevacizumab Cetuximab Panitumumab	All drugs are IgGs. Although specific data on these drugs is not known other IgG have resulted in oligohydramnios in the foetus.^34^	No experience with IV use in pregnancy in published data	Not recommended. Bevacizumab highly likely to be teratogenic and result in serious malformation given similar mechanism of action to thalidomide.^42^
Pembrolizumab		Case series suggests significant complications for use in pregnancy.^43^	Not recommended.
Dexamethasone	Well established as safe, pregnancy category A		
Metoclopramide	Well established as safe, pregnancy category A		
Ondansetron	Pregnancy category B1		

Abbreviations: TGA, Therapeutic Goods Administration; IV, intravenous; IgG, immunoglobulin; pregnancy category B1 refers to medication that has been taken by a limited number of pregnant women without an increase in risk of birth defects or other harm to the fetus.

## Surgery

Surgery is at times necessary during pregnancy, and it is the opinion of the American College of Obstetricians and gynaecologists that “pregnant woman should never be denied medically necessary surgery or have that surgery delayed regardless of trimester.”^44^ There are no consensus guidelines to aid surgical decision making related to colorectal cancer in pregnancy and it is the opinion of the authors that the best body of evidence comes from a systematic review published in 2024 of case reports of colorectal cancer in pregnancy. This series of 66 women detailed the outcomes for the 32 women who underwent surgery during their pregnancy; 78% were emergent due to presentation with an acute abdomen.^45^ Perforation occurring in the primary malignancy is rare in published reports^8^ occurring in 2.4% of colorectal cancer in pregnancy. In this setting, emergent surgery is necessary for maternal survival. An evidence-based guidelines on laparoscopy in pregnancy^46^ that analysed outcomes of emergent surgery in non-malignant conditions suggests that laparoscopy is as safe as laparotomy where appropriate surgical equipment and expertise are available. An ASCO educational book published in 2023 on cancer in pregnancy recommended that the optimum time to pursue surgery was at the start of the second trimester based on research done in gynaecological cancer^17^; there are no specific guidelines for colorectal surgery. Clearly more published data is needed.

## Conclusion

Early onset colorectal cancer is a syndrome on the rise. We have seen a 50% increase in new case referrals of patients with early-onset colorectal cancer in the last 10 years, with a preponderance for female cases in the latter two years. A large proportion (57%) of our cohort required treatment for metastatic disease. As a subset of early-onset colorectal cancer patients, pregnancy in this cohort represents a unique therapeutic dilemma. Though anxiety-provoking, delivery of chemotherapy after the first trimester of colorectal cancer can result in safe outcomes for the mother and baby. In our case series chemotherapy was not associated with any apparent birth defects in exposed fetuses. One child had significant growth issues but also had extenuating factors contributing to this clinical picture. These cases highlight the complex interplay of foetal and maternal priorities and care should ideally be provided in tertiary centres with multidisciplinary involvement.

## Data Availability

Data related to young colorectal cases in this institution is available on request.
